# Vitamin D receptor loss promotes endometrial fibrosis via autophagy-mediated epithelial–mesenchymal transition

**DOI:** 10.1016/j.gendis.2025.101803

**Published:** 2025-08-11

**Authors:** Hongtao Zhu, Bo Yang, Hui Wang, Ping Nie, Xixi Wu, Ming Yong, Xingwei Jiang, Jianguo Hu

**Affiliations:** a Department of Obstetrics and Gynecology, The Second Affiliated Hospital of Chongqing Medical University, Chongqing 400010, China; b Department of Obstetrics and Gynecology, Chongqing Medical University at Xiu Shan (People's Hospital of Xiushan County), Chongqing 409900, China; c Department of Obstetrics and Gynecology, The First Affiliated Hospital of Chongqing Medical University, Chongqing 400016, China; d Center for Reproductive Medicine, Department of Obstetrics and Gynecology, Affiliated Hospital of North Sichuan Medical College, Nanchong, Sichuan 637000, China

**Keywords:** Autophagy, EMT, Endometrial fibrosis, Intrauterine adhesion, VDR

## Abstract

Intrauterine adhesion (IUA) leads to infertility or recurrent abortion; however, its etiology and pathological mechanism remain unclear. To explore the role and mechanism of the vitamin D receptor (VDR) in the pathogenesis of IUA. We found that VDR protein expression was lower in the endometria of patients with IUA than in those of the control group. Silencing VDR in endometrial epithelial cells inhibited autophagy, promoted the epithelial–mesenchymal transition (EMT) overexpression, and increased the phosphorylation of p-MTOR, p-AKT, and p-MAPK/ERK, while its overexpression suppressed the phosphorylation of p-MTOR, p-AKT, and p-MAPK/ERK. Also, the interaction between the VDR and p62 proteins was detected. Endometrial tissue in VDR knockout mice exhibited fibrosis, reduced VDR expression, decreased ATG7, LAMP1, and LC3-II levels, and increased p62 expression; the expression of the EMT marker CDH1 decreased while that of CDH2 increased. Treatment with rapamycin reversed this process. Our data indicate that the VDR receptor is a potential marker for diagnosing and treating IUA and that vitamin D may serve as a therapeutic agent for IUA.

## Introduction

Intrauterine adhesion (IUA), also known as Asherman syndrome, is characterized by bands of fibrous tissue that form in the endometrial cavity, often in response to a uterine procedure or infection.^1^^,^^2^ The IUA damages the endometrium, causing partial or complete uterine cavity occlusion.^3^ Although recent advancements in hysteroscopic surgery have improved the diagnosis and treatment of this condition, more than half of the patients with severe IUA do not respond well to treatment.^4^ Thus, further investigations of the pathogenesis of IUA and the identification of new therapeutic targets are urgently needed.

Autophagy is a highly conserved intracellular degradation process in eukaryotes. By forming a bilayer membrane structure, it wraps and degrades damaged organelles, misfolded proteins and pathogens in the cell to maintain the homeostasis of the intracellular environment and provide nutrients and energy, playing an important role in cell survival, development, aging and disease. Autophagy can influence fibrotic diseases by promoting or inhibiting their progression.^5^^,^^6^ EMT, namely epithelial–mesenchymal transition, refers to the process in which epithelial cells gradually lose their characteristics under specific physiological and pathological conditions and obtain their characteristics. Previous studies have shown that defective autophagy contributes to endometrial EMT in patients with IUA, further aggravating endometrial fibrosis.^7^ Moreover, Peng et al identified 11 autophagy-related differentially expressed genes and 41 differentially expressed miRNAs in IUA tissue compared to normal tissues, suggesting the critical role of autophagy in IUA development.^8^

Many studies have reported that vitamin D3 reduces cell apoptosis, preserves cell survival, and decreases disease severity via the regulation of autophagy through the vitamin D receptor (VDR).^9^ A preclinical study suggested that diabetes reduces VDR expression in podocytes, impairing autophagy flux by down-regulating *Atg3* in diabetic animal models, while the activation of VDR improves glomerular autophagy flux.^10^ In addition, autophagy defects were detected in the kidneys of VDR knockout mice, and the mechanism of action related to this process seemed to be related to the AMPK pathway.^11^ Other studies have indicated that 1,25(OH)2D3 induces autophagy via the VDR-ATG16L1 signaling axis, aiding in eliminating *Klebsiella pneumoniae* infection.^12^ Based on the studies above, we hypothesized that the VDR might regulate IUA by acting on autophagy.

This study aimed to investigate the expression and function of the VDR in IUA and how the VDR regulates the pathogenesis of IUA through autophagy.

## Methods

### Endometrial tissue of normal and IUA patients

The Ethics Committee of the Second Affiliated Hospital of Chongqing Medical University approved this study, and all participants provided written informed consent ((2024)781). Written informed consents were obtained from all participants in the manuscript.

Endometrial samples were collected via endometrial biopsy from 20 patients diagnosed with IUA by hysteroscopy and 19 women of childbearing age with tubal infertility (control group) from April to October 2020.

A portion of the specimens was stored in liquid nitrogen and subsequently analyzed using qPCR and Western blot (WT), while the other portion was fixed with 4% paraformaldehyde (PFA) and analyzed via immunohistochemistry. RNA sequencing was performed on 3 normal endometrial and 3 IUA specimens to identify transcriptomic expression differences. All the subjects had abstained from estrogen and progesterone for two months before sampling.

### Next-generation sequencing technology

Total RNA was extracted from human and mouse endometrial tissue and endometrial epithelial cells following the Qiagen kit instructions (Qiagen, 74,134). The RNA quality was assessed using a 1% gel and an RNANano6000 detection kit (Agilent, 5067-1511); Shanghai Lifegenes Technology Co., Ltd. contributed to the sequencing and data analysis. Gene Ontology enrichment analysis of the differentially expressed genes was conducted using the clusterProAnalyst R package (v3.12.0). *p* values < 0.05 were considered statistically significant.

### Animals

VDR knockout mice, which were 6–8 weeks old and weighed 20–25 g, were obtained from Jiangsu Jicui Pharmaceutical Biotechnology Co., Ltd. (Cat. No. T012751). All the animals were housed in an environment with a temperature of 22 ± 1 °C, a relative humidity of 50 ± 1%, and a light/dark cycle of 12/12 h. All animal studies (including the mouse euthanasia procedure) were performed in compliance with the regulations and guidelines of Chongqing Medical University institutional animal care and conducted according to the Association for Assessment and Accreditation of Laboratory Animal Care (AAALAC) and the Institutional Animal Care and Use Committee (IACUC) guidelines. The mouse tail DNA was genotyped following the protocol from Jiangsu Jicui Pharmaceutical Biotechnology Co., Ltd. First, we bred VDR gene knockout flox homozygous mice. Identification was performed using polymerase chain reaction (PCR). PCR Primer ①: F1: CTGTCCTGGAATAGAGCAATGTGG; R1: GCAGTGACTAGGTAGCAGCAATGA; WT band: 1809 bp; Knockout (KO) band: 487 bp; PCR Primer ②: F2: TTGGTTGGTACACCTAGTGCAGG; R2: GAGCAACCTGGATGACGGTAAC; WT band: 395 bp; KO band: 0 bp (no band). Subsequently, we crossed the VDR flox homozygous mice with PgrCre homozygous mice to obtain uterine-specific VDR conditional knockout homozygous mice (VDRuterine cKO homozygotes). The control group consisted of VDR flox homozygous mice that were wild-type for Cre recombinase (CRE-negative).

### Immunohistochemistry

Human and mouse endometrial tissues were fixed with 4% PFA, embedded in paraffin, and stained with Masson’s trichrome, following the kit’s instructions. After dewaxing with xylene, gradient alcohol treatment was performed. Following antigen retrieval, 3% hydrogen peroxide was used to eliminate endogenous peroxidase, after which the samples were incubated overnight with primary antibody at 4 °C, followed by incubation with an HRP-conjugated secondary antibody for 30 min at room temperature. DAB (3,3′-Diaminobenzidine) was then used for color development. The cell nuclei were stained with hematoxylin. The staining results of the sections were quantitatively analyzed using Image-Pro Plus software. The following antibodies were applied: VDR (1:200, ab3508, Abcam, United States), LC3 (1:200, ab192890, Abcam, United States), ATG7 (1:200, ab52472, Abcam, United States), P62 (1:200, ab207305, Abcam, United States), CDH1 (1:200, ab40772, Abcam, United States), CDH2 (1:200, ab18203,Abcam, United States), and col1 (1:200, ab138492, Abcam, United States).

### Endometrial epithelial cells and overexpression vector

The human immortalized endometrial epithelial cell line (CP–H058) was obtained from Wuhan Yipu Biotechnology Co., Ltd. The cells were cultured in DMEM/F12 medium (Sigma) with 10% fetal bovine serum (FBS, HyClone) and antibiotics in a humidified atmosphere containing 5% CO_2_/95% air at 37 °C.

Genepharma (Shanghai) synthesized double-stranded oligonucleotides matching the target sequence. A VDR-overexpressing lentiviral vector (Product number HSH061736) was obtained from GeneCopoeia. The targeted sequences for human VDR and ATG7 siRNA are as follows: VDR-1: 5′-GGCUUUCACUUCAAUGCUAUG-3′, VDR-2: 5′-GGCGAAGCAUGAAGCGGAAGG-3′, ATG7: 5′-AUCAGGCACUGCUCUUGAATT-3′, and NC (negative control) siRNA: 5′-UUCUUCGAAGGUGUCACGUTT-3′. Negative control (NC) group is a cell transfected with non-targeted siRNA.

### Western blot

The Western blot procedures used were described in our previous studies.^13^ The procedures encompass protein extraction, SDS-PAGE electrophoresis, membrane transfer, immune response blocking, immune response, and chemiluminescence. The protein bands were quantified with ImageJ software. The following antibodies were applied: VDR (ab3508, 1:1000), LC3 (ab192890, 1:1000), ATG7 (ab52472, 1:1000), P62 (ab207305, 1:1000), CDH1 (ab40772, 1:1000), CDH2 (ab18203, 1:1000), and col1 (ab138492, 1:1000) from ABCM; as well as p-ERK1/2 (#4370; 1:1000), ERK1/2 (#4695; 1:1000), p-AKT (#4060; 1:1000), AKT (#4685; 1:1000), mTOR (#2972; 1:1000), and p-MTOR (#5536; 1:1000). All antibodies were from Cell Signaling Technology.

### Chromatin immunoprecipitation (ChIP) assay

ChIP detection (Cell Signaling Technology, 9003) was performed following the manufacturer’s instructions. The cross-linked chromatin was sonicated into fragments ranging from 200 to 1000 base pairs. Then, chromatin was immunoprecipitated using an anti-VDR antibody. Total RNA was extracted with a rapid extraction kit (Bioteke Corporation, RP1201). Consequently, RNA was reverse-transcribed into cDNA. Real-time quantitative PCR primers and kits for VDR amplification were obtained from GeneCopoeia, with the PCR conditions set at 95 °C for 10 s and 60 °C for 20 s. Each sample was analyzed three times. Relative mRNA quantification was conducted using the comparative threshold cycle (CT) method. This value represented gene expression using the 2^−ΔΔCT^ formula. The primers for the three loci used in the ChIP experiment are as follows: Locus 1: F: AGTGGTGGGATCTCGGCTCA; R: GGCGGATCACGAGGTCA; Locus 2: F: TGCTTATTAACCAAACTTGGGTTAA; R: AACCAATGATAATCCCAC; Locus 3: F: AGTAGCTGGGACTACAGGC; and R: TGGGCCAGGAACGTAAT. We predicted the binding sites for VDR and ATG7 using the JASPAR website. Based on the prediction scores, we selected the top three highest-scoring loci.

### Tandem mRFP-GFP-LC3 fluorescence

mRFP-GFP-tagged LC3 was used to observe autophagic flux. Endometrial epithelial cells were transfected with mRFP-GFP-LC3 and either VDR siRNA, control siRNA, or VDR-overexpressing lentivirus. After 24 h, confocal microscopy was used to analyze the distribution of mRFP-GFP-LC3 in the cells. LC3 dot quantification was performed using ImagePro Plus 6.0 software. Each experiment was conducted three times.

### Dual-luciferase assay

A luciferase reporter gene assay (Promega Corporation, E1910) was conducted as previously described.^14^ Endometrial epithelial cells were cultured in 24-well Petri dishes. After reaching 70% confluence, the cells were incubated in a fresh DMEM medium with 10% FBS. Then, the cells were transfected with the VDR-overexpressing lentiviral vector and the pGL3-ATG7 promoter plasmid (purchased from GeneCopoeia) using Endofectin-Plus (GeneCopoeia, Z01010A). Firefly luciferase activity was normalized to Renilla luciferase activity to account for transfection efficiency. Each experiment was run in triplicate.

### Statistical analysis

Data analysis was conducted using SPSS 21.0. The data are expressed as mean ± SD. Unpaired Student’s *t*-test was used for continuous variables between two groups. Multiple group comparisons were conducted using ANOVA and relevant post hoc tests. *P* < 0.05 indicated a statistically significant difference.

## Results

### VDR expression is reduced in human endometrial tissue from IUA patients

**Figure 1:**
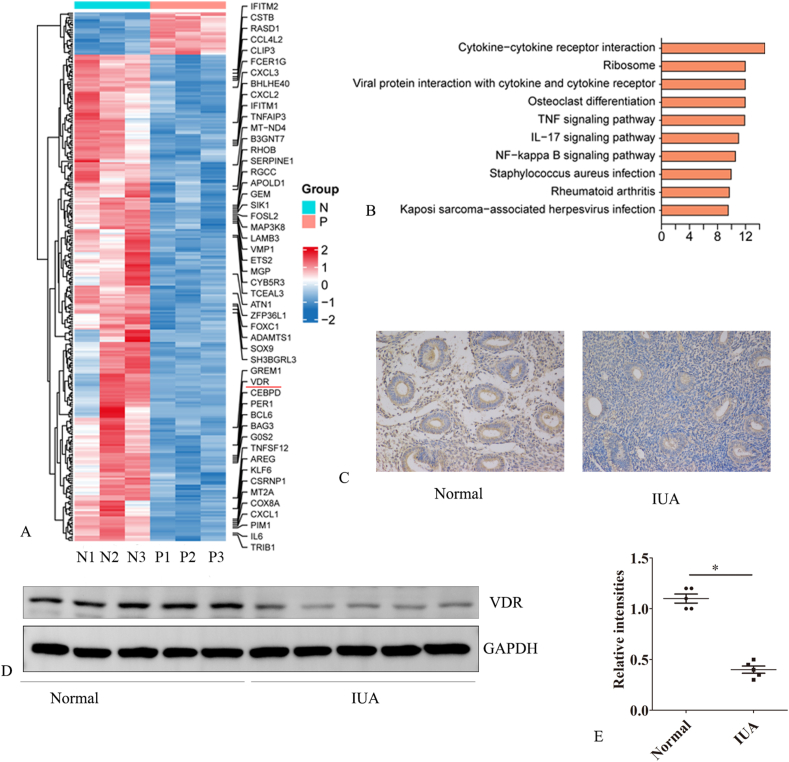
VDR expression is reduced in the endometrial tissue of patients with IUA compared to normal endometrial tissue. Next-generation transcriptome sequencing identified differential mRNA expression between normal endometrial tissue (N1–N3) and endometrial tissue from patients with IUA (P1–P3). **(A)** Heatmap. **(B)** KEGG analysis of differentially expressed genes between the normal and IUA endometrial tissues. **(C)** Immunohistochemistry was used to detect VDR expression in endometrial tissues from patients with IUA and normal endometrial tissues. **(D, E)** Western blot analysis was performed to measure VDR expression in endometrial tissues from patients with IUA (*n* = 5) and in normal endometrial tissues (*n* = 5). Student’s *t*-test was used for continuous variables between two groups (*n* = 5). Error bars indicate the standard error. The symbols ∗ and ∗∗ denote *p* < 0.05 and *p* < 0.01, respectively. Scale bar: 100 μm.

First, we analyzed the tissues of the normal endometrium (proliferative phase) and IUA endometrium (proliferative phase) collected from patients who underwent biopsy. Next-generation sequencing showed that the expression of VDR was lower in the IUA endometrium than in normal tissue (Fig. 1A). Kyoto Encyclopedia of Genes and Genomes (KEGG) analysis further suggested that the NFKB, TNF, and IL-17 pathways were associated with IUA (Fig. 1B). In addition, Western blot and immunohistochemistry showed that the expression of VDR was decreased in IUA tissues (Fig. 1C–E). Immunohistochemistry also showed that VDR was moderately expressed in endometrial stromal cells and glandular epithelial cells (Fig. 1C).

### VDR promotes autophagy in endometrial epithelial cells

**Figure 2:**
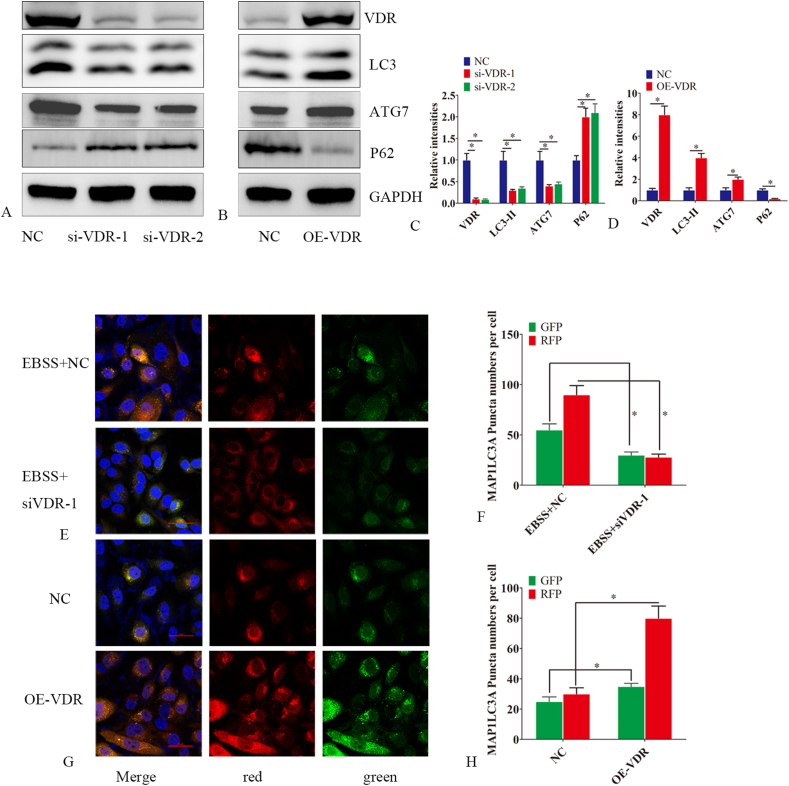
VDR enhances autophagy in endometrial epithelial cells. **(A**–**D)** Proteins were extracted from endometrial epithelial cells transfected with either a VDR siRNA (si-VDR-1 or si-VDR-2), a VDR-overexpressing lentiviral vector (OE-VDR), or a negative control siRNA (NC) for 48 h. Western blot was used to detect the expression of VDR, LC3-II, ATG7, and P62. **(E**–**H)** The mRFP-GFP-LC3 lentiviral vector was employed to measure autophagic flux in endometrial epithelial cells. Stably transfected endometrial epithelial cell lines were selected with puromycin 24 h after transfection with the mRFP-GFP-LC3 lentiviral vector. Endometrial epithelial cells, stably transfected with LC3, were subjected to NC (negative control) or VDR siRNA (si-VDR-1) transfection and treated with EBSS for 24 h. Endometrial epithelial cells stably transfected with LC3 were further transfected with either a NC or a VDR-overexpressing lentiviral vector (OE-VDR). The LC3 distribution in the cells was analyzed using laser confocal microscopy after 24 h. LC3 dots were quantified using Image-Pro Plus 6.0 software. The experiments were conducted in triplicate, and representative results are presented. Error bars indicate the standard error. The symbols ∗ and ∗∗ denote *p* < 0.05 and *p* < 0.01, respectively. Scale bar: 100 μm.

Given that autophagy participates in the occurrence and development of IUA, we next investigated the impact of VDR on autophagy in endometrial epithelial cells. VDR protein levels significantly decreased following VDR silencing (Fig. 2A–C). On the contrary, VDR overexpression significantly increased the protein level of VDR (Fig. 2B–D). Following VDR silencing, a significant decrease in the expression of LC3-II, ATG7, and p62 was observed (Fig. 2A–C), while VDR overexpression reversed this process (Fig. 2B–D). MRFP-GFP-LC3 lentivirus detection further indicated that autophagy flux decreased following VDR silencing but increased with VDR overexpression (Fig. 2E–H).

### VDR-mediated autophagy is involved in EMT

**Figure 3:**
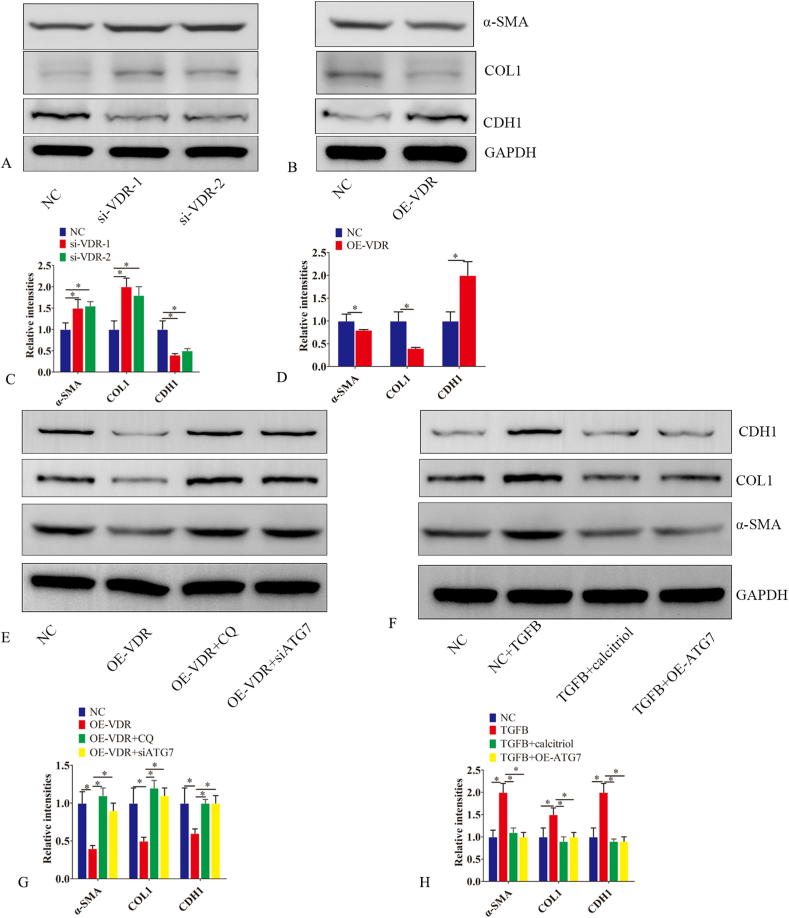
VDR-mediated autophagy is involved in EMT. **(A**–**D)** Proteins were extracted from endometrial epithelial cells transfected with VDR siRNA (si-VDR-1 and si-VDR-2), a VDR-overexpressing lentiviral vector (OE-VDR), or a NC siRNA (NC) for 48 h. Western blot was used to detect the expression of α-SMA, COL1, and CDH1. **(E, G)** Proteins were extracted from endometrial epithelial cells 48 h post-transfection with either negative control siRNA (NC), VDR overexpression lentiviral vector (OE-VDR), OE-VDR combined with 10 μM Chloroquine (OE-VDR + CQ), or OE-VDR with ATG7 siRNA (OE-VDR + ATG7 siRNA). Western blot analysis was conducted to measure the expression levels of α-SMA, COL1, and CDH1. **(F, H)** Endometrial epithelial cells were transfected with negative control siRNA (NC), NC with TGFβ1 (10 ng/ml), TGFβ1 (10 ng/ml) with calcitriol (10 nM), and TGFβ1 (10 ng/ml) with an ATG7-overexpressing plasmid (OE-ATG7). Proteins were extracted 48 h later. Western blot analysis was conducted to measure the expression levels of α-SMA, COL1, and CDH1. The experiments were conducted in triplicate, and representative results are presented. Error bars indicate the standard error. The symbols ∗ and ∗∗ denote *p* < 0.05 and *p* < 0.01, respectively.

Previous studies have shown that defective autophagy contributes to endometrial EMT in patients with IUA, further aggravating endometrial fibrosis.^7^ To further investigate the role of VDR in endometrial epithelial cells (EECs)-EMT, we performed VDR knockdown and overexpression in EECs and subsequently analyzed EMT-related indices. In EECs transfected with si-VDR, CDH2, col1, and α-SMA protein levels were up-regulated compared to those in the control group, whereas CDH1 expression was down-regulated (Fig. 3A–C). Unlike the down-regulation of VDR, its overexpression did not protect EECs from EMT (Fig. 3B–D). The cytoprotective effect of VDR overexpression was nullified in CQ-pretreated EECs or with ATG7 silencing, indicating that VDR inhibits EEC-EMT via autophagy induction (Fig. 3E–G).

We further discussed the role of calcitriol in EEC-EMT. After being pretreated with TGFB for 24 h, EECs were incubated with calcitriol (10 nM) for another 24 h. Calcitriol or ATG7 overexpression reversed TGFB-induced EEC-EMT (Fig. 3F–H).

### VDR regulates autophagy via the MAPK/ERK and PI3K/AKT/MTOR pathways

**Figure 4:**
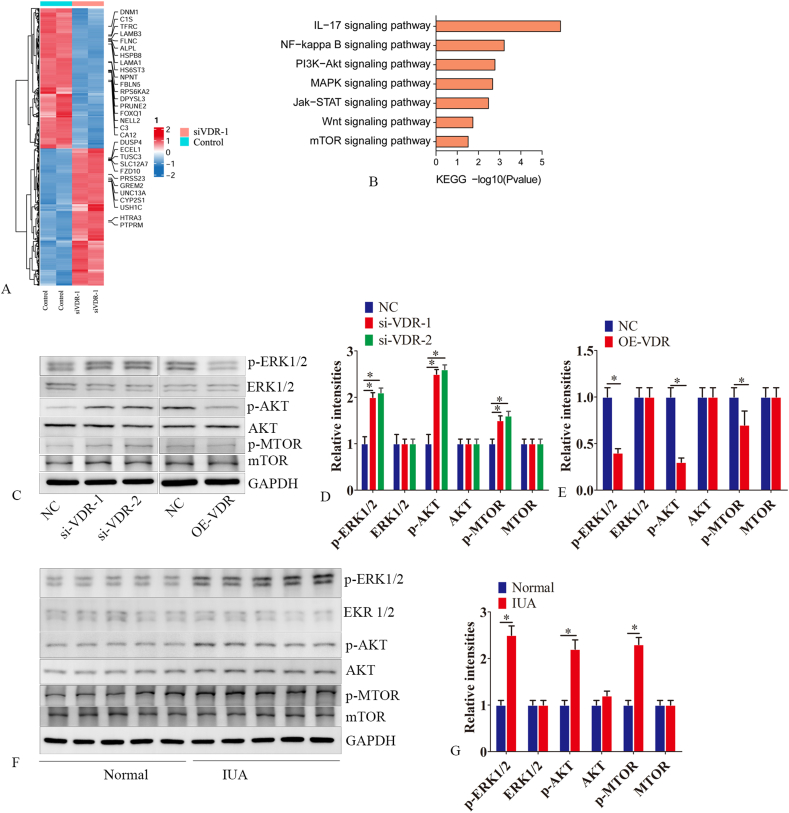
VDR regulates autophagy via the MAPK/ERK and PI3K/AKT/MTOR pathways. Endometrial epithelial cells were transfected with either negative control siRNA or VDR siRNA, followed by RNA extraction after 48 h. Transcriptomic differential expression was detected using next-generation sequencing. **(A)** Heatmap. **(B)** KEGG analysis of differentially expressed mRNAs between the VDR silencing and negative control groups. **(C**–**E)** Endometrial epithelial cells were transfected with negative control siRNA (NC), si-VDR-1, si-VDR-2, OE-VDR (VDR-overexpressing lentiviral vector), or NC (VDR-overexpressing control). Proteins were extracted 48 h later. Western blot analysis was performed to detect the expression levels of p-ERK1/2, ERK1/2, p-AKT, AKT, p-mTOR, and mTOR. **(F, G)** Western blot analysis was conducted to measure the levels of p-ERK1/2, ERK1/2, p-AKT, AKT, p-mTOR, and mTOR in endometrial tissues from patients with IUA and those with normal endometrial tissues. The experiments were conducted in triplicate, and representative results are presented. Error bars indicate the standard error. The symbols ∗ and ∗∗ denote *p* < 0.05 and *p* < 0.01, respectively.

Sequencing of VDR-silenced cells revealed abnormal expression of the MAPK/ERK and PI3K/AKT-mTOR pathways (Fig. 4A, B). Given that the MAPK/ERK and PI3K/AKT-MTOR pathways regulate autophagy, we next examined alterations in MAPK/ERK, AKT, and MTOR following VDR overexpression or silencing. VDR silencing increased the phosphorylation of p-MTOR, p-AKT, and p-MAPK/ERK; conversely, VDR overexpression suppressed the phosphorylation of p-MTOR, p-AKT, and p-MAPK/ERK (Fig. 4C–E).

We also analyzed the expression of p-ERK1/2, p-AKT, and p-MTOR in IUA and normal endometrial tissues. The p-AKT, p-ERK1/2, and p-MTOR levels were elevated in IUA endometrial tissues compared to normal endometrium (Fig. 4F, G).

### ATG7 is a direct target of VDR

**Figure 5:**
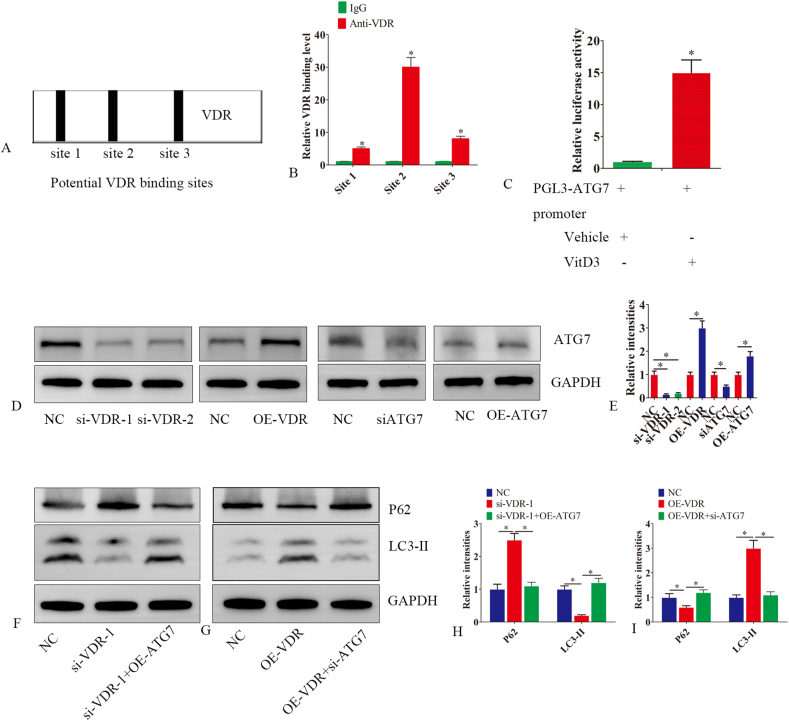
ATG7 is a direct target of VDR. **(A)** We predicted the binding sites for VDR and ATG7 using the JASPAR website. Based on the prediction scores, we selected the top three highest-scoring loci. **(B)** Chip experiments were used to determine the direct binding of VDR to the ATG7 promoter. **(C)** ChIP assays were performed with endometrial epithelial cells cultured in the presence or absence of VitD3. Immunoprecipitation was performed with anti-VDR. VDR binding to the ATG7 promoter was analyzed via qRT-PCR and was represented as a percentage of the input. **(D, E)** Endometrial epithelial cells were transfected with negative control siRNA (NC), si-VDR-1, si-VDR-2, siATG7, OE-ATG7 or OE-VDR (VDR-overexpressing lentiviral vector), or NC (VDR-overexpressing control). Proteins were extracted 48 h later. Western blot analysis was performed to detect the expression levels of ATG7 and GAPDH. **(F–I)** Endometrial epithelial cells were transfected with negative control siRNA (NC), si-VDR-1, si-VDR-1+OE-ATG7, OE-VDR or OE-VDR + si-ATG7, or NC (VDR-overexpressing control). Proteins were extracted 48 h later. Western blot analysis was performed to detect the expression levels of P62 and LC3-II. The experiments were conducted in triplicate, and representative results are presented. Error bars indicate the standard error. The symbols ∗ and ∗∗ denote *p* < 0.05 and *p* < 0.01, respectively.

VDR, as a transcription factor, regulates downstream gene expression by binding to the promoter. Bioinformatics analysis using JASPAR identified three potential VDR-binding sites in the promoter region of ATG7 (Fig. 5A). By using a chromatin immunoprecipitation (ChIP) assay, we determined that VDR interacted with the ATG7 promoter. Our findings indicated that VDR bound to all three potential binding sites of the ATG7 promoter, with the second site exhibiting the strongest binding affinity (Fig. 5B). Moreover, the results of the luciferase reporter assay confirmed that calcitriol positively regulated ATG7 expression (Fig. 5C). VDR silencing reduced ATG7 protein expression in endometrial epithelial cells. The overexpression of VDR produced the opposite effect (Fig. 5D, E). VDR silencing reduced LC3-I/II conversion, while ATG7 overexpression partially mitigated this effect. VDR overexpression increased LC3-I/II conversion, while ATG7 silencing partially inhibited this effect. These data indicated that the VDR-regulated autophagy was dependent on ATG7 (Fig. 5F–I).

### VDR uterine conditional knockout mice showed an endometrial fibrosis phenotype

**Figure 6:**
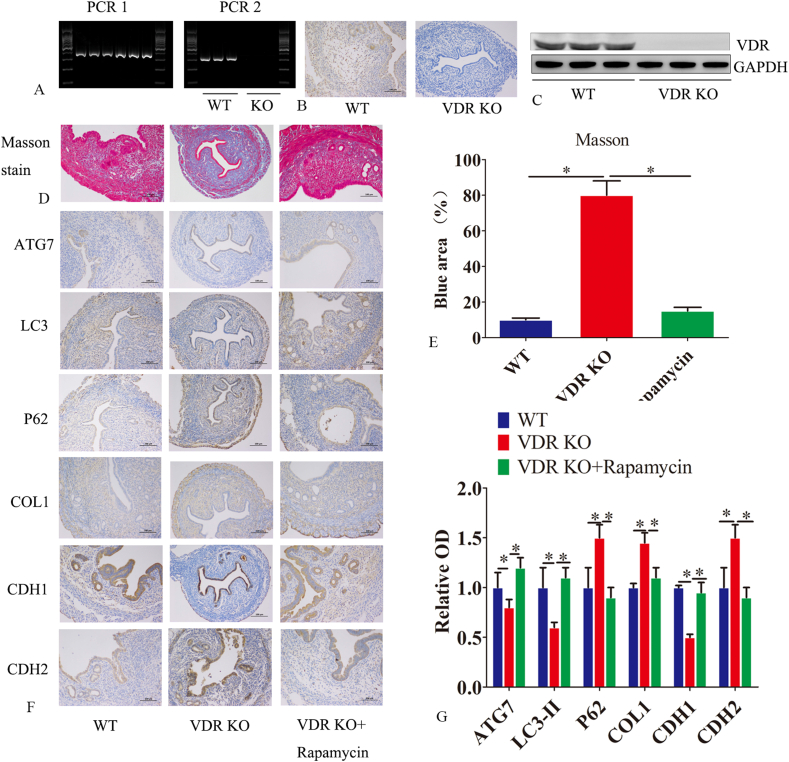
VDR uterine conditional knockout mice showed an endometrial fibrosis phenotype. **(A)** PCR was used to identify wild-type (WT) and VDR knockout homozygous mice. **(B)** Immunohistochemistry was employed to assess VDR expression in both WT and VDR knockout (KO) homozygous mice. **(C)** Western blot analysis was conducted to assess VDR expression in wild-type (WT) and VDR knockout (KO) homozygous mice. The experiment comprised three groups, namely, WT, VDR-KO, and VDR KO + rapamycin, with 5 mice per group. Rapamycin (1 mg/kg per mouse) was administered via intraperitoneal injection 3 times a week for 4 weeks. **(D, E)** Masson staining was used to assess fibrosis levels across the three groups. **(F, G)** Immunohistochemistry was employed to assess the expression of ATG7, LC3, P62, COL1, CDH1, and CDH2 in the endometrial tissues across the three groups of mice. The experiments were conducted in triplicate, and representative results are presented. Student’s *t*-test was used for continuous variables between two groups (*n* = 3). Error bars indicate the standard error. The symbols ∗ and ∗∗ denote *p* < 0.05 and *p* < 0.01, respectively. Scale bar: 100 μm.

In order to confirm the relationship between VDR and endometrial fibrosis, we constructed VDR gene knockout mice. PCR, immunohistochemistry, and WB confirmed the significant down-regulation of VDR expression in the VDR-KO homozygotes (Fig. 6A–C). In VDR knockout mice, vacuolar changes were observed in endometrial glandular epithelial cells, accompanied by a reduction in endometrial glands and damage to glandular epithelial cells (Fig. 6D). Masson staining further showed obvious fibrosis in the endometrial glandular epithelium and stroma of VDR-KO mice compared with those of WT mice (Fig. 6D). In the VDR-KO mice, the expression of VDR, ATG7, LAMP1, and LC3-II decreased, while the expression of p62 increased. The expression of the EMT marker CDH1 decreased while that of CDH2 increased. After treatment with rapamycin, the opposite effect was observed.

## Discussion

This study observed decreased VDR expression in the endometrial tissues of patients with IUA. Its low expression reduced autophagy and EMT transformation in endometrial epithelial cells. In VDR knockout (VDR-KO) mice, fibrotic states and impaired autophagy were observed in the endometria of C57VDRKO mice. After the autophagy activator rapamycin was administered, the endometrial fibrosis status of C57 VDR-KO mice improved.

Vitamin D has been associated with various fibrotic diseases, including lung, kidney, and liver fibrosis. Vitamin D deficiency can cause pulmonary fibrosis.^15^ For example, preclinical testing found that VDR knockout mice exhibit damage to the glomerular epithelium and a fibrotic phenotype in kidney tissue^11^

Intestinal fibrosis is a complication associated with Crohn’s disease, and VDR expression abnormalities are strongly linked to intestinal fibrosis.^16^ In this study, next-generation transcriptome sequencing, immunohistochemistry, and Western blot analysis revealed lower VDR expression in the endometrial tissues of patients with IUA than in the endometrial tissues collected from women of childbearing age with tubal infertility.

Endometrial fibrosis is a pathological manifestation of IUA. To verify the role of VDR in endometrial fibrosis, we generated VDR knockout mice. We found that VDR-KO mice showed fibrotic endometria compared to wild-type mice. Our findings indicate that decreased VDR expression in the endometrium leads to endometrial fibrosis and suggest that VDR may be used as a diagnostic marker for IUA.

Vitamin D is intricately linked to autophagy. The VDR/Atg3 axis regulates autophagy and inhibits the development of diabetic nephropathy.^10^ Also, vitamin D enhances autophagy and reduces macrophage lipid accumulation through the VDR/PTPN6 signaling pathway. The mechanism involves the activation of the MAPK1 (mitogen-activated protein kinase 1) signaling pathway by VDR.^9^ Another study found that VDR agonists activate autophagy in Kaposi’s sarcoma cells by inhibiting the PI3K/Akt/mTOR signaling pathway.^17^ Li et al found that VDR activates the MAPK signaling pathway, inducing autophagy in renal tubular epithelial cells.^11^ Our study demonstrated that VDR activates autophagy in endometrial epithelial cells. We also discovered that the VDR protein interacts with the P62 protein and regulates autophagy via the MAPK/ERK and PI3K/AKT/mTOR signaling pathways. IUA patients exhibit deficient endometrial autophagy, and its activation can inhibit fibrosis.^7^ Therefore, we speculate that the lack of autophagy caused by decreased VDR in the endometrium is a pathogenesis of uterine adhesives.

EMT is essential for normal embryogenesis. However, the EMT process is beneficial to promote the fibrosis in tissues and organs. Targeting EMT inhibition is a potential therapeutic approach for remodeling tissue and organ fibrosis.^18^, ^19^, ^20^ Many studies have confirmed that autophagy deficiency can promote the EMT process. Autophagy inducers can inhibit EMT progression, making them potential therapeutic targets for fibrotic diseases.^21^, ^22^, ^23^ For example, a previous study found that astragaloside IV can alleviate renal fibrosis by inhibiting EMT and activating autophagy.^24^ Another study found that autophagy deficiency in the endometria of patients with IUA is linked to elevated EMT, and autophagy activation inhibits EMT in endometrial epithelial cells.^7^ A previous study found that VDR regulates defective autophagy in renal tubular epithelial cells in streptozotocin-induced diabetic mice via the AMPK pathway.^11^ This study found that VDR activates autophagy and inhibits the EMT process in endometrial epithelial cells. The primary mechanism involves the interaction between VDR and p62 and the regulation of the MAPK/ERK and PI3K/AKT/MTOR signaling pathways. In the endometria of VDR-KO mice, we also found that endometrial autophagy decreased and EMT increased. The absence of VDR in the endometria of patients with IUA appears to reduce autophagy, thereby promoting EMT and endometrial fibrosis. Vitamin D had potential as a therapeutic agent for IUA.

This study is the first to identify decreased VDR expression in the endometria of patients with IUA. We found that reduced VDR expression in the endometria of patients with IUA facilitates endometrial fibrosis via autophagy-mediated EMT. Our data suggest that vitamin D may be potentially used as a treatment for IUA.

## CRediT authorship contribution statement

**Hongtao Zhu:** Writing – review & editing, Writing – original draft, Visualization, Funding acquisition, Formal analysis, Data curation, Conceptualization. **Bo Yang:** Validation, Supervision. **Hui Wang:** Supervision, Resources, Project administration, Methodology, Investigation. **Ping Nie:** Software, Methodology, Investigation. **Xixi Wu:** Visualization, Validation, Methodology. **Ming Yong:** Visualization, Validation, Supervision, Software, Data curation. **Xingwei Jiang:** Project administration, Formal analysis, Conceptualization. **Jianguo Hu:** Writing – original draft, Resources, Methodology, Investigation, Funding acquisition, Conceptualization.

## Funding

This work was supported by the Sichuan Science and Technology Program (China) (No. 2022NSFSC1357), the Doctoral Initiation Fund Project (Natural Science) (China) (No. CBY21-QD23), the Kuanren Talents Program of The Second Affiliated Hospital of Chongqing Medical University (China) (No. kryc-yq-2104, kryc-yq-2222), the Senior Medical Talents Program of Chongqing for Young and Middle-aged (China), and Chongqing Science and Technology Program (China) (No. CSTB2024TIAD-KPX0038).

## Conflict of interests

The authors declare that they have no competing interests.

## References

[1] Park S., Cho Y., Kim H.S. (2023). Mesonephric-like adenocarcinoma of the uterine corpus: clinicopathological and prognostic significance of L1 cell adhesion molecule (L1CAM) over-expression. Anticancer Res.

[2] Li L.N., Li X.D., Du J. (2023). The effect of aspirin on uterine arterial blood flow and endometrium in moderate and severe intrauterine adhesion after transcervical resection of adhesion: a systematic review and meta-analysis. J Matern Fetal Neonatal Med.

[3] Zhao X., Sun D., Zhang A. (2022). Uterine cavity parameters evaluated by hysteroscopy can predict the live birth rate for intrauterine adhesion patients. Front Med.

[4] Liu A.Z., Zhao H.G., Gao Y., Liu M., Guo B.Z. (2016). Effectiveness of estrogen treatment before transcervical resection of adhesions on moderate and severe uterine adhesion patients. Gynecol Endocrinol.

[5] Wang Y., Ping Z., Gao H. (2024). LYC inhibits the AKT signaling pathway to activate autophagy and ameliorate TGFB-induced renal fibrosis. Autophagy.

[6] Livingston M.J., Zhang M., Kwon S.H. (2024). Autophagy activates EGR1 *via* MAPK/ERK to induce FGF2 in renal tubular cells for fibroblast activation and fibrosis during maladaptive kidney repair. Autophagy.

[7] Zhou Z., Wang H., Zhang X. (2022). Defective autophagy contributes to endometrial epithelial-mesenchymal transition in intrauterine adhesions. Autophagy.

[8] Peng X., Zhu Y., Wang T., Wang S., Sun J. (2023). Integrative analysis links autophagy to intrauterine adhesion and establishes autophagy-related circRNA-miRNA-mRNA regulatory network. Aging (Albany NY).

[9] Kumar S., Nanduri R., Bhagyaraj E. (2021). Vitamin D3-VDR-PTPN6 axis mediated autophagy contributes to the inhibition of macrophage foam cell formation. Autophagy.

[10] Wang B., Qian J.Y., Tang T.T. (2021). VDR/Atg3 axis regulates slit diaphragm to tight junction transition *via* p62-mediated autophagy pathway in diabetic nephropathy. Diabetes.

[11] Li A., Yi B., Han H. (2022). Vitamin D-VDR (vitamin D receptor) regulates defective autophagy in renal tubular epithelial cell in streptozotocin-induced diabetic mice *via* the AMPK pathway. Autophagy.

[12] Tang J., Gu L., Luo J., Luo H., Zeng Q., Jiang Y. (2022). 1, 25(OH)_2_D_3_ promotes the elimination of *Klebsiella pneumoniae* infection by inducing autophagy through the VDR-ATG16L1 pathway. Int Immunopharmacol.

[13] Zhang Z., Hu J. (2024). DKK1 loss promotes endometrial fibrosis *via* autophagy and exosome-mediated macrophage-to-myofibroblast transition. J Transl Med.

[14] Hu J., Meng Y., Zhang Z. (2017). MARCH5 RNA promotes autophagy, migration, and invasion of ovarian cancer cells. Autophagy.

[15] Ma D., Peng L. (2019). Vitamin D and pulmonary fibrosis: a review of molecular mechanisms. Int J Clin Exp Pathol.

[16] Yu M., Wu H., Wang J. (2021). Vitamin D receptor inhibits EMT *via* regulation of the epithelial mitochondrial function in intestinal fibrosis. J Biol Chem.

[17] Suares A., Tapia C., González-Pardo V. (2019). VDR agonists down regulate PI3K/Akt/mTOR axis and trigger autophagy in Kaposi’s sarcoma cells. Heliyon.

[18] Shu D.Y., Butcher E., Saint-Geniez M. (2020). EMT and EndMT: emerging roles in age-related macular degeneration. Int J Mol Sci.

[19] Guo L.P., Chen L.M., Chen F., Jiang N.H., Sui L. (2019). Smad signaling coincides with epithelial-mesenchymal transition in a rat model of intrauterine adhesion. Am J Transl Res.

[20] Zhao G., Li R., Cao Y. (2020). ΔNp63α-induced DUSP4/GSK3β/SNAI1 pathway in epithelial cells drives endometrial fibrosis. Cell Death Dis.

[21] Kong D., Zhang Z., Chen L. (2020). Curcumin blunts epithelial-mesenchymal transition of hepatocytes to alleviate hepatic fibrosis through regulating oxidative stress and autophagy. Redox Biol.

[22] Wu P., Wang X., Yin M. (2024). ULK1 mediated autophagy-promoting effects of rutin-loaded chitosan nanoparticles contribute to the activation of NF-κB signaling besides inhibiting EMT in Hep3B hepatoma cells. Int J Nanomed.

[23] Huang Z., Kaller M., Hermeking H. (2023). CRISPR/Cas9-mediated inactivation of miR-34a and miR-34b/c in HCT116 colorectal cancer cells: comprehensive characterization after exposure to 5-FU reveals EMT and autophagy as key processes regulated by miR-34. Cell Death Differ.

[24] Li D., Liu Y., Zhan Q. (2023). Astragaloside IV blunts epithelial-mesenchymal transition and G2/M arrest to alleviate renal fibrosis *via* regulating ALDH2-mediated autophagy. Cells.

